# Negative pressure wound therapy for surgical wounds healing by secondary intention is not cost-effective

**DOI:** 10.1093/bjs/znaf077

**Published:** 2025-05-06

**Authors:** Pedro Saramago, Athanasios Gkekas, Catherine E Arundel, Ian C Chetter, the SWHSI-2 Trial Investigators, Belen Corbacho Martin, Catherine Hewitt, Andrew Mott, Samantha Swan, David Torgerson, Jacqueline Wilkinson, Sabeen Zahra, Jane Blazeby, Rhiannon Macefield, Stephen Dixon, Josie Hatfield, Angela Oswald, Jo Dumville, Matthew Lee, Thomas Pinkney, Nikki Stubbs, Lyn Wilson

**Affiliations:** Centre for Health Economics, University of York, York, UK; York Trials Unit, University of York, York, UK; York Trials Unit, University of York, York, UK; Faculty of Health Sciences, University of Hull, Hull, UK; Hull York Medical School, Hull, UK; Hull University Teaching Hospitals NHS Trust, Hull, UK

## Abstract

**Background:**

Negative pressure wound therapy (NPWT) has been used in clinical practice for surgical wounds healing by secondary intention (SWHSI), despite limited evidence regarding its clinical effectiveness and cost-effectiveness. The aim of this study was to evaluate the cost-effectiveness of NPWT for SWHSI, compared with standard dressings, from the perspective of the UK healthcare system.

**Methods:**

An economic model was used to extrapolate the effectiveness results of a meta-analysis over a patient’s lifetime and estimate the costs and outcomes (quality-adjusted life-years (QALYs)) of NPWT and standard dressings. The probability of NPWT being cost-effective was estimated, with extensive scenario analyses conducted to evaluate the robustness of results and the degree of uncertainty.

**Results:**

On average, NPWT was associated with higher costs and marginally higher QALYs than standard dressings. The cost difference was mainly driven by the additional intervention costs associated with NPWT. The estimated probability of NPWT being cost-effective was <30%. There was considerable uncertainty in the findings, driven largely by uncertainty in the estimated pooled relative effect from the meta-analysis. Results were robust to different scenario analyses.

**Conclusion:**

No evidence was found demonstrating that NPWT was a cost-effective alternative to standard dressings for SWHSI.

## Teaser

An economic evaluation was implemented to compare negative pressure wound therapy (NPWT) with usual care dressings for surgical wounds healing by secondary intention (SWHSI). No evidence was found demonstrating that NPWT was a cost-effective alternative to usual care dressings for SWHSI.

## Introduction

After an operation, most wounds are closed by apposing the wound edges—‘healing by primary intention’. If closure is not possible, or if a primarily closed wound breaks down, the wound may be left open to heal from the bottom up, through formation of granulation tissue—‘healing by secondary intention’.

Surgical wounds healing by secondary intention (SWHSI) are complex and common, with an estimated UK prevalence of 4.1 per 10 000 population^1^. SWHSI treatment poses a significant healthcare burden, given the time taken for wound healing (median of 86 (95% c.i. 75 to 130) days), as well as frequent wound infection (32.1%), hospital readmission (24.7%), and further surgical procedures (16.8%)^2^. Depending on the treatment used, costs have been estimated to be between £1501 and £2383 per patient per month^3,4^.

Negative pressure wound therapy (NPWT) is a commonly utilized treatment option for SWHSI. NPWT involves a wound dressing system that applies a subatmospheric pressure to the wound surface, removing infective materials and exudate, reducing oedema, and promoting perfusion, cellular proliferation, and granulation tissue formation, thus creating an environment more conducive to wound healing^1^.

The use of NPWT to theoretically augment SWHSI healing has increased dramatically in recent years^2^. However, robust evidence to support the clinical effectiveness and cost-effectiveness of NPWT is limited. A Cochrane review failed to identify any rigorous RCT evidence regarding the clinical effectiveness of NPWT in this population^3^. A more recent non-randomized cohort study, with extensive adjustment for confounders, found that NPWT was not clinically effective or cost-effective for SWHSI^4^. Additionally, in other patient groups (for example open traumatic wounds), where its use is common practice, recent high-quality evidence questions the clinical effectiveness and cost-effectiveness of NPWT^5^.

There was therefore a need to establish RCT evidence to definitively assess the clinical effectiveness and cost-effectiveness of NPWT as a treatment to augment SWHSI healing. This was supported by the UK National Institute for Health and Care Excellence (NICE) recommendations for research^6,7^. The National Institute for Health and Care Research (NIHR) funded the SWHSI-2 trial, which evaluated the clinical effectiveness and cost-effectiveness of NPWT *versus* standard dressings in patients with SWHSI^8,9^.

The SWHSI-2 clinical results found no clear evidence that NPWT reduced the time to healing compared with standard dressings (HR 1.08 (95% c.i. 0.88 to 1.32), *P* = 0.47) and no clear evidence that NPWT reduced complications (infection, readmission, and other surgical procedures). A within-trial economic analysis, considering only evidence from the SWHSI-2 trial, found NPWT to marginally increase quality-adjusted life-years (QALYs) and increase healthcare costs over the 12-month study interval^8^. These results were not statistically significant. There are, however, significant limitations of within-trial analysis to inform healthcare decisions on the cost-effectiveness of health technologies^10–12^. Current guidance highlights the need for a comprehensive consideration of all relevant evidence^13^.

This paper presents a comprehensive economic model evaluation, conducted alongside the SWHSI-2 trial, including external evidence, to determine the cost-effectiveness of NPWT compared with standard dressings as an SWHSI treatment and to provide a robust foundation for decision-making^9^.

## Methods

The cost-effectiveness of alternative SWHSI treatments was evaluated using a Markov model, a mathematical tool typically used in health economics to assess the costs and outcomes of a healthcare intervention over time. This Markov model compared NPWT with standard dressings for SWHSI over a patient’s lifetime, that is using a time horizon of 30 years. Costs are expressed in UK pounds sterling at 2022 prices and outcomes in terms of QALYs, a composite health outcome measure that captures length of life and health-related quality-of-life (HRQoL) of patients. This economic analysis followed NICE guidance^13^, and considered the UK National Health Service (NHS) perspective, with costs and outcomes discounted over time.

**Fig. 1:**
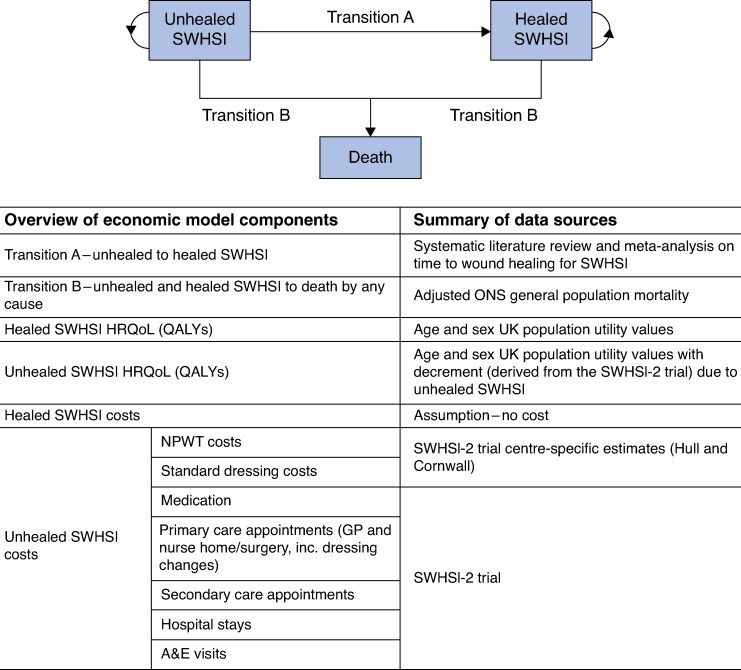
Economic model structure for SWHSI SWHSI, surgical wounds healing by secondary intention; ONS, Office for National Statistics; HRQoL, health-related quality of life; QALYs, quality-adjusted life-years; NPWT, negative pressure wound therapy; GP, general practitioner; inc., including; A&E, accident and emergency.

The economic model structure, components, and data sources are presented in *Fig. 1*. The structure was based on clinical input regarding the key health states of patients with SWHSI: healed SWHSI, unhealed SWHSI, and death. The model considered three main health state transitions: from unhealed to healed SWHSI (where the key event is time to wound healing), from unhealed SWHSI to death, and from healed SWHSI to death. The model structure, inputs, and results were validated by SWHSI-2 clinical researchers and advisors to the project.

All patients started in the unhealed SWHSI health state, with the occurrence of wound healing and transition to the healed SWHSI state tracked over time. The effectiveness of NPWT and standard dressings was established within the economic model by how fast patients moved from the unhealed to the healed health state. In accordance with standard care, patients in the standard dressings group within the model were assumed to be treated with standard dressings while unhealed. Patients in the NPWT group were assumed to be treated with NPWT for a proportion of time and with standard dressings for the remaining time. Patients remaining in the unhealed and healed SWHSI health states had specific HRQoL and health resource use and costs associated with them over time. For transitions from unhealed and healed SWHSI states to death, the economic model considered the probability of patients dying over time.

The model was implemented in RStudio, version 2024.04.0 Build 735^14^. The R code is available from the corresponding author upon request.

### Economic model parameters, sources, and assumptions

*Table 1* provides a comprehensive list of all of the economic model parameters considered, as well as details regarding the estimates used for each parameter and information sources.

**Table 1:** Summary of the economic model parameters

Parameter	Value	Source
**Specification of the patient population**		
Baseline age (years, mean(s.e.))	62.9(12.6)	SWHSI-2 trial^8^
Wound area >25 cm^2^, %	37.7	SWHSI-2 trial^8^
Treatment location, % inpatient (*versus* outpatient)	83.1	SWHSI-2 trial^8^
Tissue involvement, % skin and subcutaneous tissue loss (*versus* skin loss)	78.4	SWHSI-2 trial^8^
SWHSI history, %	16.8	SWHSI-2 trial^8^
**Transition probabilities from unhealed wound to healed**		
Pooled relative treatment effectiveness (posterior distribution), HR (95% c.i.)	1.07 (0.82,1.71)	Bayesian meta-analysis synthesizing six studies from the literature and SWHSI-2 data^8^
**Transition to death**		
Mortality risk	Adjusted general population mortality; 84 deaths (12.2%) (66 unhealed and 18 healed); SMR = 13.3 (additional mortality risk for an SWHSI patient compared with general population mortality)	ONS^21^ (2022) SWHSI-2 trial^8^
**HRQoL**		
** **General population utilities for healed patients (health-state utility)	Utility values of the UK population	Ara and Brazier^31^ (2010)
Monthly utility decrement in the unhealed wound health state (health-state utility)	0.0095(0.004)	Mixed-effect model using SWHSI-2 trial^8^
**Costs and resource use**		
Time on treatment with NPWT (days)	46.6(4.12)	SWHSI-2 trial^8^
NPWT daily cost (£)	30.61(0.18)	Centre-specific costings (Hull and Cornwall), assuming a combination of canister and dressing sizes and types, and based on the breakdown of dressings used and averaging the price of advanced dressings
Standard dressings daily cost (£)	4.17(0.11)	Centre-specific costings (Hull and Cornwall), assuming a combination of canister and dressing sizes and types, and based on the breakdown of dressings used and averaging the price of advanced dressings
Monthly health resources while healed	0	Assumption
**Monthly primary care health resources consumed while unhealed (resources assumed to be treatment dependent)**		
GP surgery appointments (NPWT)	0.11(0.03)	SWHSI-2 trial^8^
GP surgery appointments (standard dressings)	0.13(0.05)	SWHSI-2 trial^8^
GP home appointments (NPWT)	0.05(0.01)	SWHSI-2 trial^8^
GP home appointments (standard dressings)	0.07(0.04)	SWHSI-2 trial^8^
Nurse surgery appointments (NPWT)	1.39(0.22)	SWHSI-2 trial^8^
Nurse surgery appointments (standard dressings)	1.26(0.19)	SWHSI-2 trial^8^
Nurse home appointments (NPWT)	3.65(0.35)	SWHSI-2 trial^8^
Nurse home appointments (standard dressings)	3.89(0.45)	SWHSI-2 trial^8^
Dressing change appointments (NPWT)	14.67(2.44)	SWHSI-2 trial^8^
Dressing change appointments (standard dressings)	12.82(2.24)	SWHSI-2 trial^8^
**Monthly secondary care health resources consumed while unhealed (resources assumed to be treatment independent)**		
Hospital outpatient appointments (diabetic foot clinic)	0.24(0.02)	SWHSI-2 trial^8^
Hospital outpatient appointments (podiatry)	0.06(0.01)	SWHSI-2 trial^8^
Hospital outpatient appointments (specialty dressing clinic)	0.01(0.001)	SWHSI-2 trial^8^
Hospital outpatient appointments (vascular, colorectal, or plastics)	0.01(0.001)	SWHSI-2 trial^8^
Hospital admissions without an overnight stay	0.01(0.001)	SWHSI-2 trial^8^
Hospital inpatient nights	0.74(0.13)	SWHSI-2 trial^8^
Accident and emergency service visits	0.02(0.001)	SWHSI-2 trial^8^
Medication cost while unhealed (£)	6.18(0.65)	SWHSI-2 trial^8^

Values are mean(s.e.) unless otherwise indicated. SWHSI, surgical wounds healing by secondary intention; SMR, standardized mortality ratio; ONS, Office for National Statistics; HRQoL, health-related quality of life; NPWT, negative pressure wound therapy; GP, general practitioner.

The characteristics of the SWHSI patient population within the economic model have been drawn from the SWHSI-2 trial population. To inform the effectiveness of treatments for SWHSI, a systematic literature review (SLR) was undertaken (see the *Supplementary material*). Data from studies identified in the SLR in summary format and from studies with available patient-level data (SWHSI-2 trial) were synthesized in a meta-analysis (WinBUGS code: *Supplementary material*^15^). The pooled HR from the meta-analysis on time to wound healing defined the probability of moving to the healed SWHSI health state. A higher pooled HR estimate in the meta-analysis relates to higher effectiveness for the healing of SWHSI.

Further SLRs (see the *Supplementary material*) were conducted to identify evidence on: HRQoL, costs and resource use, and mortality. Evidence of interest included: changes to HRQoL/utility for a patient whose wound heals; resource use and cost data incurred by patients with an unhealed wound; and data regarding the relationship between an unhealed wound and increased mortality risk.

The SLR on HRQoL identified no eligible studies (*Supplementary material*). HRQoL for SWHSI patients was therefore derived from SWHSI-2 trial data. In the SWHSI-2 trial, the EuroQol-Five Dimensions-Five Levels (EQ-5D-5L) questionnaire was used as a validated instrument to evaluate HRQoL^16,17^. This questionnaire was completed by trial participants at baseline and 3, 6, and 12 months, and valued through mapping to the corresponding Three Levels questionnaire, as recommended by NICE. EQ-5D index scores range from 1 (full health) to 0 (death), with negative values representing a HRQoL worse than death. Mean(s.e.) EQ-5D index scores, conditional on SWHSI healing status, were estimated for each time point (see the *Supplementary material*). The decrement of having an unhealed SWHSI was estimated via regression modelling.

The SLR for healthcare resource use and costs identified a retrospective UK cohort study of patients with unhealed surgical wounds that estimated the mean resource use and costs per patient. Available data were, however, considered incompatible with the economic model requirements, as a reduced number of resource items were reported in the retrospective UK cohort study, with no consumption by treatment arm detailed^18^. Thus, the economic model was informed by SWHSI-2 trial resource use data. Resource use data included data on primary care service use (for example general practitioner (GP) surgery or home visits, and practice nurse surgery or home visits) and medication use (for example analgesics, antibiotics, non-steroidal anti-inflammatory drugs (NSAIDs), and others), which were collected via a participant questionnaire at baseline and 3-, 6-, and 12-month follow-up. Secondary care health resource use data (for example hospital admissions with/without an overnight stay, hospital outpatient appointments, and accident and emergency visits) were collected using an investigator-completed questionnaire. It was assumed that, once healed, an SWHSI patient would be discharged and no further healthcare resources would be used and, thus, no costs would be incurred by the health system. Unit costs of healthcare services were collected from the Unit Costs of Health and Social Care (PSSRU and NHS Reference Costs)^19^ and medication costs were collected from the British National Formulary (see the *Supplementary material*)^20^.

The SLR on mortality identified no eligible studies (*Supplementary material*). General population mortality was obtained from the Office for National Statistics (ONS)^21^. Although it was considered that a SWHSI may not directly affect patients’ mortality, the characteristics of this population may imply a higher mortality rate than that of the general population. Thus, an excess mortality for SWHSI patients was derived from the SWHSI-2 trial data.

A summary of the key economic model assumptions is shown in *Table 2*.

**Table 2:** Key economic model assumptions

Assumption	Description
Treatment groups	Assumed the treatment to which SWHSI-2 trial patients were randomized to, that is an intention-to-treat analysis.
Patient population	Assumed that the SWHSI-2 trial population reflected the population of interest for this evaluation.
Patients’ HRQoL	To derive the quality-of-life decrement of being in the unhealed SWHSI health state, it was assumed that patients’ HRQoL was conditional only on their healing status and not on other patient characteristics.
Time on standard dressings and on NPWT	Patients in the standard dressings group were assumed to be treated with standard dressings while unhealed. Patients in the NPWT group were assumed to be treated with NPWT for a proportion of time while unhealed and with standard dressings for the remaining time, conditional on the time on NPWT treatment being shorter/longer than the estimated time to healing.
Healed SWHSI costs	Assumed that, once healed, an SWHSI patient would be discharged and no further healthcare resources would be used and, thus, no costs would be incurred by the health system.
Mortality	Assumed that the probability of death is equivalent for patients in the unhealed and healed SWHSI health states. Assumed that the mortality of the SWHSI population is higher than the mortality of the general population, adjusted for age and sex.

SWHSI, surgical wounds healing by secondary intention; HRQoL, health-related quality of life; NPWT, negative pressure wound therapy.

### Cost-effectiveness

Total QALYs and costs per patient over a 30-year time horizon were derived. Total costs were divided into healthcare-related costs and intervention-related costs. Costs and QALYs were used to derive the net monetary benefit (NMB) for each treatment^22^, the incremental NMB, and the probability of NPWT being cost-effective^23^. NMB compares the costs and benefits of an intervention in monetary terms. Higher NMB and positive and higher incremental NMB estimates imply better value for money^10^.

To account for the uncertainty in the evidence informing the economic model and its assumptions, scenario analyses were performed to evaluate the sensitivity of the economic model results to implemented key assumptions, namely: on SWHSI-2 trial treatment allocation—NPWT received at any point *versus* no-NPWT (rather than randomized treatment); on treatment effectiveness evidence; on mortality estimates; and on health resource use.

How much higher the clinical effectiveness of NPWT was required to be, compared with standard dressings, to alter the cost-effectiveness results was also analysed.

## Results

### SWHSI healing and mortality

The SLR on effectiveness evidence identified 18 studies, one systematic review^24^, and one Cochrane Review^3^. From these, six studies were deemed eligible to inform the meta-analysis comparing the effect of NPWT and standard dressings on time to SWHSI healing^25–30^. The pooled HR from the meta-analysis was aligned with the SWHSI-2 clinical results, finding no clear evidence that NPWT reduced the time to healing compared with standard dressings (HR 1.07 (95% c.i. 0.82 to 1.71)).

**Fig. 2:**
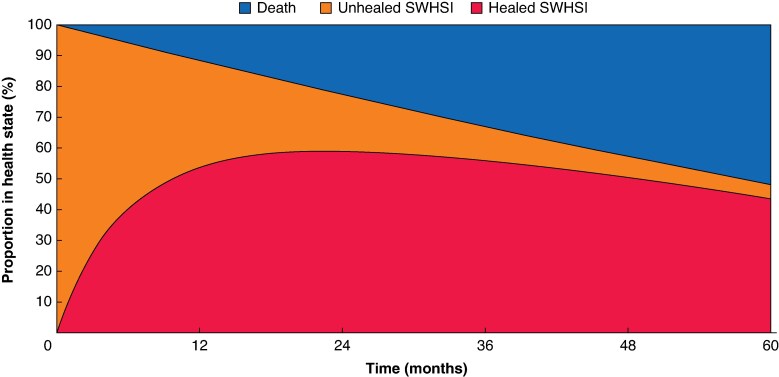
Movement through the economic model health states for the initial 5 years for both the NPWT group and the standard dressings group SWHSI, surgical wounds healing by secondary intention; NPWT, negative pressure wound therapy.

*Figure 2* shows the proportion of patients in each health state for the initial 5 years (that is 60 monthly cycles) of the economic model. The flow of patients for standard dressings and NPWT was virtually identical, thus results are shown for the whole population. At the start of the model, all patients were in the unhealed state. At 12 months, for patients with standard dressings, 55.0% were expected to be healed (compared with 55.3% with NPWT), 32.7% were expected to still be unhealed (compared with 32.4% with NPWT), and 12.4% were expected to be deceased (irrespective of treatment). The proportion of healed patients increased over time, with 91.0% of alive patients who received standard dressings estimated to be healed at 5 years, compared with 91.4% for those who received NPWT. The mortality of this SWHSI population was estimated to be much higher than the mortality of the general population (standardized mortality ratio (SMR) of additional mortality risk compared with age-adjusted general mortality risk of 13.3, that is the probability of dying in this SWHSI population was 13.3 times higher than that in the general population). At 5 years, 52.5% of patients were estimated to be deceased, rising to 100% after 22.8 years—a reminder that the economic model starting age is 62.9 years.

### Costs and outcomes of treatments for SWHSI

*Table 3* shows the lifetime costs and outcomes associated with each treatment. The QALY gains for NPWT compared with standard dressings (mean of 0.01 (95% c.i. −0.02 to 0.05)), reflect the results from the meta-analysis, with differences between health benefits with NPWT and standard dressings being small and uncertain.

**Table 3:** Total costs and effects of treatments for SWHSI

	NPWT	Standard dressings
Total QALYs	3.77 (3.63,3.87)	3.76 (3.62,3.86)
**Total costs (£)**	92 436 (70 470,114 516)	89 642 (73 038,110 037)
Healthcare costs	73 881 (53 603,94 824)	75 940 (59 773,95 890)
Intervention costs	18 554 (14 804,21 971)	13 702 (12 349,15 101)
Incremental costs (*versus* standard dressings) (£)	2794 (−11 916,13 559)	−
Incremental QALYs (*versus* standard dressings)	0.01 (−0.02,0.05)	−
**£20 000/QALY gained cost-effectiveness threshold (willingness-to-pay)**		
NMB (£)	−17 018 (−39 208,5672)	−14 414 (−34 318,3032)
Incremental NMB (£)	−2604 (−13 901,12 853)	
Probability of NPWT being cost-effective, %	28.9	
**£30 000/QALY gained cost-effectiveness threshold (willingness-to-pay)**		
NMB (£)	20 692 (−2053,44 054)	23 200 (2159,40 880)
Incremental NMB (£)	−2509 (−14 029,13 323)	
Probability of NPWT being cost-effective, %	29.5	

Values are mean(s.e.) unless otherwise indicated. SWHSI, surgical wounds healing by secondary intention; NPWT, negative pressure wound therapy; QALYs, quality-adjusted life-years; NMB, net monetary benefit.

Before healing, healthcare resources were lower for NPWT than for standard dressings, except for dressing change appointments, which were higher (mean(s.e.) of 12.82(2.24) appointments/month for standard dressings *versus* 14.67(2.44) appointments/month for NPWT). Results also show that healthcare costs were estimated to be lower, on average, for NPWT than for standard dressings (mean per patient healthcare costs of £73 881 for NPWT *versus* £75 940 for standard dressings) over a patient’s lifetime (note that costs were only accrued when unhealed).

The mean(s.e.) duration of treatment was 46.6(4.1) days for patients in receipt of NPWT and 164.3(4.7) days for patients in receipt of standard dressings. At a mean daily cost of £4.17 for standard dressings and £30.61 for NPWT, the mean per patient intervention costs were substantially higher for NPWT than for standard dressings while unhealed (£18 554 *versus* £13 702 respectively).

### Cost-effectiveness results

**Fig. 3:**
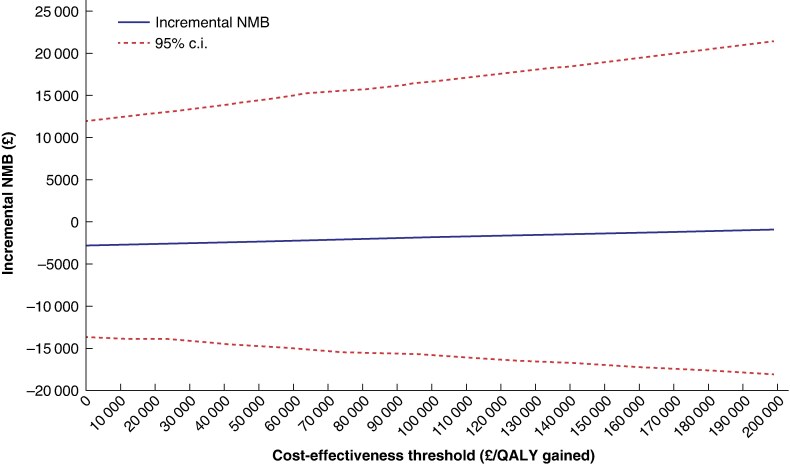
Incremental NMB for a range of cost-effectiveness thresholds NMB, net monetary benefit; QALY, quality-adjusted life-year.

*Table 3* reports incremental costs and effects (in the QALY format), as well as the NMB for each treatment, the incremental NMB, and the probability of NPWT being cost-effective at cost-effectiveness threshold/opportunity cost values of £20 000/QALY gained and £30 000/QALY gained. These are the usual threshold values used by NICE in decisions to approve or reject health technologies. Standard dressings had the highest mean NMB values, indicating that standard dressings are cost-effective compared with NPWT. Moreover, the negative incremental NMB values (−£2604 at the £20 000/QALY gained cost-effectiveness threshold and −£2509 at the £30 000/QALY gained cost-effectiveness threshold) indicate that NPWT is not cost-effective, which is supported by the low estimates for the probability of NPWT being cost-effective (28.9% and 29.5% respectively). Furthermore, in *Fig. 3*, incremental NMB of NPWT over standard care is shown for a range of opportunity cost values, with the mean incremental NMB not being positive over the entire range, meaning that NPWT is likely not to be good value for money for the UK NHS compared with standard dressings (also see the *Supplementary material*).

### Scenario analysis

Scenario analyses explored the sensitivity of the model findings. Irrespective of the scenario, the key message, of NPWT not being considered cost-effective when compared with standard dressings, prevailed (*Supplementary material*). For NPWT to be considered cost-effective, NPWT would need to increase the probability of wound healing by 16% relative to standard dressings.

## Discussion

This economic model evaluated the cost-effectiveness of NPWT compared with standard dressings in SWHSI patients from the perspective of the UK healthcare system.

The analysis found that, overall, NPWT is more expensive than standard dressings (mean per patient total costs over their lifetime of £92 436 for NPWT and £89 642 for standard dressings). NPWT has higher intervention costs compared with standard dressings (mean per patient intervention costs while unhealed of £18 554 for NPWT and £13 702 for standard dressings), but lower healthcare costs. The expected NMB was higher for standard dressings than for NPWT, indicating that NPWT is not a cost-effective treatment for SWHSI patients. Results were uncertain, although robust to alternative assumptions.

Economic model estimates of clinical effectiveness were informed by pooled SLR evidence, which stemmed from a Cochrane review^3^. The meta-analysis developed made best use of all available RCT-based effectiveness evidence on NPWT for SWHSI, as it considered data at the patient level, when available. The meta-analysis estimates highlight considerable uncertainty over the pooled relative treatment effect, reflecting the fact that many of the included studies had small sample sizes. However, although uncertainty in the evidence base was reflected via a wide confidence interval, the effect of including low-quality studies was less transparent, with five (of six) studies deemed to be at moderate or high risk of bias.

Limited external data were identified, with model parameters primarily drawn from the SWHSI-2 trial data. Uncertainty was prevalent in several of the economic model parameters, which was addressed via scenario analyses that evaluated how sensitive results and assumptions of the economic model were, mainly by testing the inclusion of evidence from a previous cohort study^4^. A scenario that pooled patients who received NPWT at baseline and who received NPWT at a later stage during the SWHSI-2 trial produced similar findings to the intention-to-treat results^8^. Irrespective of the scenarios tested, the assumption of including a range of different standard dressings as a single comparator for this evaluation was always present and may be a source of unexplored heterogeneity.

Another limitation lies with the model assumption that healed SWHSI patients do not generate any present or future costs to the healthcare system, that is they are discharged and do not consume further health resources, representing zero costs. This implies that the estimated total costs may be underestimated. However, it was not possible to obtain any relevant cost estimates for SWHSI-2 patients once healed, given the follow-up interval of 1 year, thus the authors encourage the conduct of longitudinal studies to explore longer-term healthcare costs in this population.

To inform the HRQoL of patients in the unhealed SWHSI health state, HRQoL was assumed to depend solely on SWHSI healing status and not on other patient characteristics. The effect of healing is small, but statistically significant; estimated mean(s.e.) monthly decrement of 0.0095(0.004) in quality of life for those who are unhealed compared with those who are healed. This estimate is half that for the previous cohort (0.018)^2^, although the characteristics of the previous cohort are less severe (for example, younger patients and smaller and less deep wounds). This potential SWHSI patient heterogeneity was also considered in scenario analyses, not changing key findings.

The SWHSI-2 trial planned to recruit participants from a range of surgical fields; however, this was a significant challenge, resulting in approximately 90% of the SWHSI included in the study arising from vascular surgeries; thus, the economic findings may be more relevant to this subgroup of patients.

It should also be noted that the SWHSI-2 trial assessed use of NPWT in relation to time to healing only. NPWT use differs between surgical specialties, with some specialties using NPWT for wound or exudate management, rather than for healing. Conclusions regarding NPWT effectiveness for these purposes are not covered by the study or this analysis.

Although limited evidence exists in the literature on the cost-effectiveness of NPWT for SWHSI, the findings of the present study align with previous publications using observational data, which found that NPWT was not cost-effective compared with standard dressings, with little uncertainty over this result^6,27^. Given that existing evidence is UK based, the generalizability of the findings to other health systems outside the UK is uncertain.

The effectiveness evidence on time to SWHSI healing found no clear evidence of a difference in favour of NPWT when compared with standard dressings. Results suggest that NPWT should probably not be considered a first-line treatment for SWHSI, particularly when the wound is located on the lower limb. NPWT and standard dressings offer similar HRQoL benefits, but NPWT is more expensive due to higher intervention costs. The probability of NPWT being cost-effective was low, although results remain uncertain.

## Collaborators

Belen Corbacho Martin, Professor Catherine Hewitt, Andrew Mott, Samantha Swan, Professor David Torgerson, Jacqueline Wilkinson, Sabeen Zahra (University of York, York, UK); Professor Jane Blazeby, Rhiannon Macefield (University of Bristol, Bristol, UK); Stephen Dixon, Josie Hatfield, Angela Oswald (Hull University Teaching Hospitals NHS Trust, Hull, UK); Professor Jo Dumville (University of Manchester, Manchester, UK); Mr Matthew Lee (Sheffield Teaching Hospitals NHS Foundation Trust); Professor Thomas Pinkney (University of Birmingham, Birmingham, UK); Nikki Stubbs (Leeds Community Healthcare NHS Trust, Leeds, UK); Lyn Wilson (Mid Yorkshire Teaching NHS Trust, Wakefield, UK).

## Supplementary Material

znaf077_Supplementary_Data

## Data Availability

Anonymized data sets generated and analysed during the present study will be stored in a publicly available open research repository (https://osf.io/echxv). Data will be made available via this repository after completion of analysis and subsequent publication. Sharing of these anonymized data is covered by original participant consent for the SWHSI-2 trial, which permits sharing of data to support future research via sharing anonymously.
