# Supplementary material for: Negative pressure wound therapy for surgical wounds healing by secondary intention is not cost-effective
Source: Br J Surg. 2025 May 6;112(5):znaf077. doi: 10.1093/bjs/znaf077 (PMC12053490; doi:10.1093/bjs/znaf077)
Supplement: znaf077_Supplementary_Data [file znaf077_supplementary_data.docx]

**Electronic Supplementary Materials**

**Section 1 - Systematic literature review (SLR) on effectiveness and meta-analysis**

To maximise available effectiveness evidence to inform the decision analytic model, a systematic review was undertaken to identify relevant randomised controlled trials evaluating the effectiveness of negative pressure wound therapy (NPWT) as a treatment for SWHSI.

***Search strategy***

Effectiveness evidence for NPWT as a treatment for SWHSI has been previously collated in a Cochrane review.^1^ Given the established robustness of Cochrane reviews, we opted to utilise and re-run this search strategy. An initial search was run in MEDLINE, with date restrictions applied to identify articles published from 1^st^ May 2015 (date of the Cochrane search) until 14^th^ June 2022. A further search was then conducted on 17^th^ October 2023, to identify any subsequent publications.

***Study Selection and Data Extraction***

Titles and abstracts retrieved from the searches were downloaded into COVIDENCE (Veritas Health Innovation, Melbourne, Australia) and de-duplicated. The remaining titles and abstracts were independently screened by one of four reviewers against pre-specified inclusion and exclusion criteria.

Studies were eligible for inclusion if they:

- Included patients with a SWHSI;
- Included NPWT as either the intervention or control treatment;
- Assessed time to wound healing as an outcome;
- Used a randomised controlled trial (RCT) design.

Studies were ineligible for inclusion if they:

- Were protocol, abstract or review publications. Where these were identified and potentially relevant, we sought to check that the associated RCT had not been omitted from the database search.

Full text copies were obtained for those articles deemed to be meeting the inclusion criteria and were independently reviewed by one of four reviewers. Any disagreements or uncertainties were discussed and resolved with input from a fifth reviewer. Using a standardised data extraction form, data extraction was completed and a 10% sample of extraction by each reviewer was independently checked for consistency.

***Analytical sample of relevant studies***

Effectiveness evidence from RCTs was obtained from identified studies and assessed for inclusion in the meta-analysis. The final meta-analysis contained data from seven RCTs on the relative effectiveness of NPWT and standard dressings for the treatment of SWHSI. Data for one of the seven included RCTs (SWHSI-2 trial) full individual patient-level data (IPD) was available (686 participants) which included time to healing or censoring for each participant, together with other individual-level characteristics such as treatment location, wound area, tissue involvement and a history of SWHSI. For the remaining RCTs (corresponding to 784 participants in total), aggregate data on the number of healed wounds were extracted from the source review alongside information regarding treatment type, number of participants allocated to each treatment group, participant’s age at baseline, mean duration of follow-up (if this was not stated, trial duration was used), wound location, duration, size and sign of infection. Table S1 describes the data available.

***Statistical Bayesian meta-analysis model***

Data were synthesised by assuming that the time to wound healing underlying both aggregate data (AD) and the IPD was described by the same parametric distribution, following the approach described in Saramago et al.^2^ For the IPD, where time to healing (under censoring) was observed for each SWHSI-2 participant – these data could directly inform the distribution of the time to healing (the likelihood). For the AD, by assuming a binomial likelihood, the number of participants healed was used to inform the probability of participants being healed. In turn, the probability of participants being healed was related (algebraically) to the common distribution of time to healing taking into account the duration of follow-up in each study (analogous to the synthesis model defined by Saramago et al.^2^ This approach allowed all wound healing data (proportion of wounds healed and time to wound healing) to be defined as time to wound healing, thus the measure of effectiveness used to report pooled findings of the meta-analysis was the hazard ratio (HR).

The use of IPD allowed standard fully parametric survival distributions to be fit to the time to wound healing data. The following time to event distributions were used: exponential, Weibull, Gompertz, log-normal, log-logistic and generalised gamma. The parametric survival modelling of the IPD study was implemented using regression survival analysis that allowed for baseline covariate adjustment. Baseline covariates considered were treatment location, wound area, tissue involvement and a history of SWHSI. All relative treatment effects were presented as HRs. Alternative synthesis modelling specifications were implemented to assess the between-study heterogeneity, and the inclusion of baseline covariates. Goodness of fit was assessed using the deviance information criterion (DIC).^3^

All evidence synthesis were conducted from a Bayesian perspective, using WinBUGS software version 1.4.3 (MRC Biostatistics Unit, Cambridge, UK. URL: https://www.mrc-bsu.cam.ac.uk/bugs/winbugs/contents.shtml).

**Table S1 Analytic sample**

| **Study** | **Country** | **Treatment** | **Wound location (majority)** | **Follow up (weeks)** | **Number patients** | **Baseline mean age (years)** | **Baseline mean wound size (cm2)** | **Number healed (%)** | **Evidence available format** |
| --- | --- | --- | --- | --- | --- | --- | --- | --- | --- |
| Acosta *et al.* 2013 ^4^ | Sweden | NPWT | Groin | 17.3 | 5 | 74 | - | 4 (80%) | AD |
|  |  | StdDres | Groin | 17.3 | 5 | 74 | - | 3 (60%) |  |
| Armstrong *et al.* 2005 ^5^ | USA | NPWT | Foot | 16 | 77 | 57 | 22.3 | 31 (40%) | AD |
|  |  | StdDres | Foot | 16 | 85 | 60 | 19.2 | 25 (29%) |  |
| Ulas -Biter *et al.* 2014 ^6^ | Netherlands | NPWT | Pilonidal sinus | 52 | 24 | 23 | 15.3 | 24 (100%) | AD |
|  |  | StdDres | Pilonidal sinus | 52 | 25 | 29 | 14.2 | 25 (100%) |  |
| Chiang *et al.* 2017 ^7^ | New Zealand | NPWT | Foot | 52 | 18 | 61 | 38.8 | 9 (50%) | AD |
|  |  | StdDres | Foot | 52 | 18 | 62 | 32.9 | 7 (39%) |  |
| Monsen *et al.* 2014^8^ | Monsen | NPWT | Groin (infected) | 17.3 | 10 | 71 | 13 | 9 (90%) | AD |
|  |  | StdDres | Groin (infected) | 17.3 | 10 | 73 | 20.5 | 7 (70%) |  |
| Seidel *et al.* 2020^9^ | Germany, Belgium and Netherlands | NPWT | Abdomen | 6 | 256 | 66 | - | 27 (11%) | AD |
|  |  | StdDres | Abdomen | 6 | 251 | 66 | - | 31 (12%) |  |
| SWHSI-2 | United Kingdom | NPWT | Foot | 52 | 349 | 63 | 26.5 | 210 (60%) | IPD |
|  |  | StdDres | Foot | 52 | 337 | 63 | 27.6 | 199 (59%) |  |

***Evidence quality assessment***

The Cochrane Risk of Bias tool (version 2) was used to assess risk of bias for the relevant outcome (time to wound healing) applying all domains of the tool.^10^ One of the reviewers who completed the data extraction independently assessed the risk of bias of each study with assessment independently verified by a second reviewer.

Of the six publications identified for inclusion in the literature review,^4-9^ alongside the SWHSI-2 trial data, three studies were deemed to be at high risk of bias,^4, 7, 8^ two with some concerns,^5, 6^ and one at low risk of bias.^9^ The primary cause of bias related to Domain 5 ‘Selection of the reported result’.

**Figure S1 RoBv2 effectiveness evidence quality assessment.**


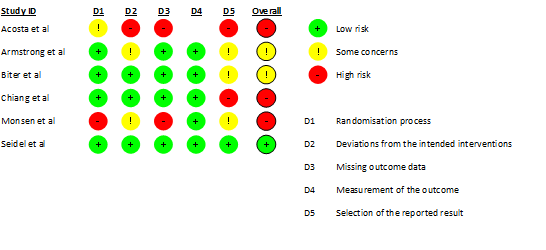


**Section 2 - WinBUGS code**

This code relates to the random-effects with covariates evidence synthesis model assuming time to healing follows a log-normal distribution. The model is here described in a generic form for it to be easy for the user to modify and adapt to specific applications. Four data elements are required to fit the complete model: two containing constants for AD and IPD, one for the study at IPD level and one for the AD evidence. All data should be loaded before the model is compiled. Because of size and agreements of use, the original data sets are not included in their entirety, but a couple of lines of data are supplied for each study/data combination for illustration purposes.

model {

*### Part 1: Model for IPD ###*

for (i in 1:n.subjects1) {

*#Log-normal likelihood for IPD*

t.obs[i] ~ dlnorm(mu[i],tau)I(t.cens[i],)

*#Model for IPD*

mu[i] <- mu1 + delta[TNP1[i]] * (1 - equals(TNP1[i], baseline[i])) + beta_cov * cov[i]

*#Residual deviance for IPD*

p1.hat[i] <- 1 - phi((log(t.obs[i]) - mu[i]) * sqrt(tau))

p1[i] <- 1 - phi((log(t.obs[i]) - mean(mu[])) * sqrt(tau))

dev1[i] <- (p1.hat[i] - p1[i]) * (p1.hat[i] - p1[i])

}

*#Vague priors for IPD 1*

mu1~dnorm(0,1.0E-6)

resdev1 <- sum(dev1[])

*#Mean of Log Hazard Ratios*

for (l in 1:n.arms1) {

md[l] <- d[treat1[l]] - d[baseline1[l]]

delta[l] ~ dnorm(md[l], prec)

}

*### Part 2: Model for AD ###*

for (i in 1:n.agg.arm) {

*#Binomial likelihood for AD*

r[i]~dbin(pa[i],n[i])

*#Model for AD*

pa[i] <- 1 - phi((log(a.time[i]) - mu.a[i]) * sqrt(tau))

mu.a[i] <- mu1.a[a.s[i]] + delta[i + n.arms1] * (1-equals(a.treat[i],a.base[i]))

*#Mean of Log Hazard Ratios*

delta[i + n.arms1] ~ dnorm(md.ad[i], prec)

md.ad[i] <- d[a.treat[i]] - d[a.base[i]]

*#Residual deviance for AD*

pa.obs[i] <- r[i] / n[i]

dev.ad[i] <- (pa.obs[i] - pa[i]) * (pa.obs[i] - pa[i])

}

*#Sum of residual deviance for AD*

resdev.ad <- sum(dev.ad[])

*#Vague priors for AD*

for(j in 1:n.agg.trials) {

mu1.a[j]~dnorm(0,1.0E-6)

}

*#Vague priors for IPD covariate*

beta_cov ~ dnorm(0,1.0E-6)

*### Model for combining all estimates of treatment effect ###*

*#Total residual deviance*

totresdev <- resdev1 + resdev.ad

*#Vague priors*

tau ~ dgamma(1.0,0.0001)

d[1]<-0

for (k in 2:max.treat) {

d[k] ~ dnorm(0,1.0E-6)

hr[k]<- exp(d[k])

}

tau1~dunif(0,5)

tau1.sq<-tau1*tau1

prec<-1/(tau1.sq)

}

*### Dataset 1: Constants to define for IPD evidence###*

*# Number of participants in IPD 1 #*

list(n.subjects1 = 430,

*# Number of treatments being evaluated*

max.treat = 2,

*# treatment indicator*

treat1=c(1,2)

*# baseline treatment indicator*

baseline1=c(1,1),

*# number of treatment arms*

n.arms1=2)

*### Dataset 2: Constants to define for AD evidence###*

*# Number of AD studies #*

list(n.agg.trials = 6,

*# Number of AD study arms #*

n.agg.arms = 12)

*### Dataset 3: IPD ###*

| TNP1[] | baseline[] | t.obs[] | t.cens[] | cov1[] |
| --- | --- | --- | --- | --- |
| 1 | 1 | 3.71 | 0 | 1.95 |
| 1 | 1 | 2.33 | 0 | 1.94 |
| 2 | 1 | NA | 11.99 | 2.49 |
| ... | ... | ... | ... | ... |
| ... | ... | ... | ... | ... |

END

# TNP1 = treatment arm (coded 1,2), baseline = reference treatment code,

# t.obs = time to event in months (under censoring), t.cens = time of censoring in months,

# cov1 = continuous covariate of interest (R+)

*### Dataset 4: AD evidence ###*

| a.s[] | a.treat [] | r[] | n[] | a.base[] | a.time[] |
| --- | --- | --- | --- | --- | --- |
| 1 | 1 | 25 | 85 | 1 | 4 |
| 1 | 2 | 31 | 77 | 1 | 4 |
| ... | ... | ... | ... | ... | ... |
| ... | ... | ... | ... | ... | ... |

END

# a.s = study number, a.treat = treatment arm code (coded from 1 to number of treatments),

# r = number of events in trial arm, n = number of patients in trial arm,

# a.base = reference treatment code, a.time = follow-up time of trial (in months)

*### Initial values, either need specifying or generating for the below scalars and vectors ###*

list(delta = c(0,0,0,0,0, 0,0,0,0,0, 0,0), d = c(NA,0), mu1 = -1, mu1.a = c(0,0,0,0,0), tau = 1, tau1 = 1, beta_cov = 0)

**Section 3 – Assessment of time to healing in SWHSI-2 and Bayesian meta-analysis results**

***Assessment of time to healing in SWHSI-2***

SWHSI-2 total number of participants=686

NPWT (allocation 1) =349 (202 (57.9%) healed over FU); NPWT median time to healing 187 days (95%CI 169 - 226)

Usual care dressings (allocation 0) =337 (196 (58.1%) healed over FU); Usual care dressings median time to healing 196 days (95%CI 158 - 213)


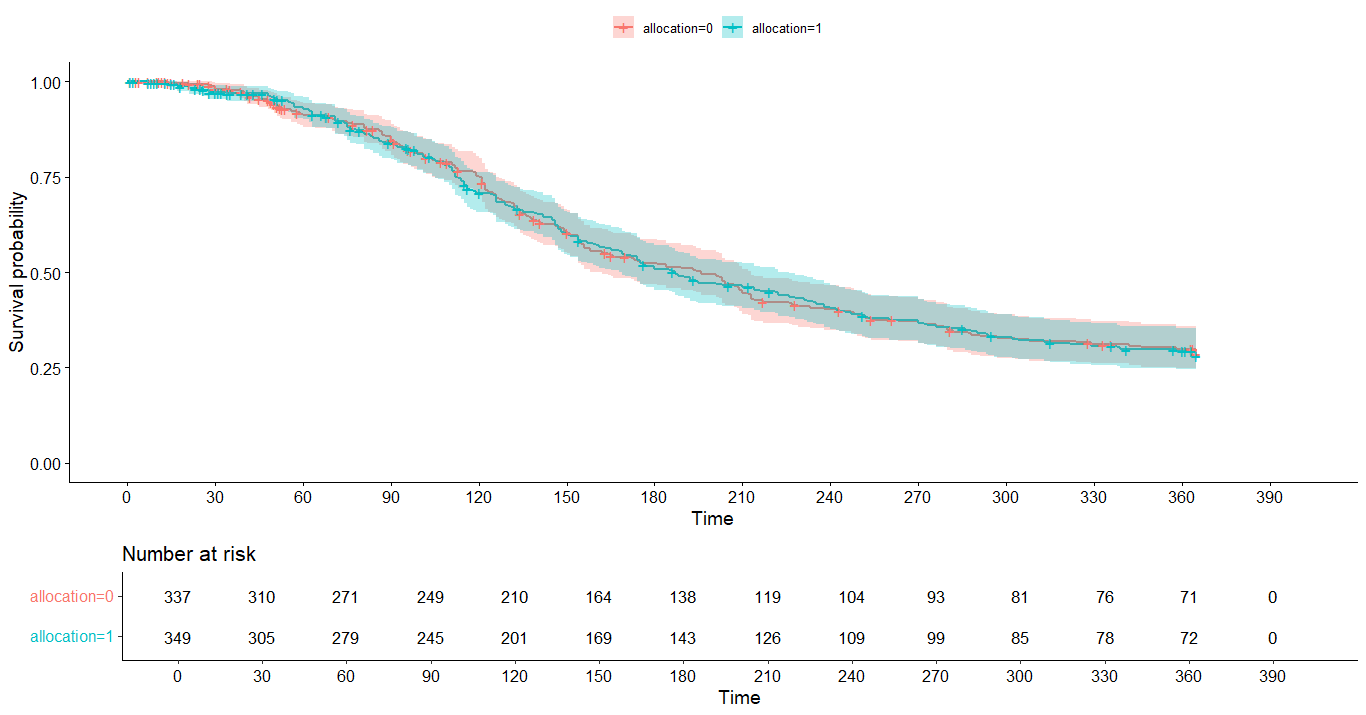


Semi-parametric survival model on SWHSI-2 time to wound healing data (Cox PH only treatment adjusted): **Cox PH: HR= 1.01 (SE=0.10) p=0.93**

Goodness of fit of fully parametric survival models on SWHSI-2 time to wound healing data (only treatment adjusted):

| **Time to wound healing parametric model** | **AIC** | **BIC** |
| --- | --- | --- |
| Exponential PH | 5358 | 5367 |
| Weibull PH | 5302 | 5320 |
| Gompertz | 5347 | 5365 |
| Log-logistic AFT | 5258 | 5276 |
| Log-normal AFT | **5255** | **5268** |
| Generalised Gamma | 5257 | 5275 |
| PH, proportional hazards; AFT, accelerated failure time | | |

Log-normal identified as best (lower AIC/BIC and visual fit)

**Log-normal: HR=0.98 (95%CI 0.84-1.15)**

When performing temporal extrapolation, the Log-normal survival model predicts the proportion of healed wounds to be: at 1y= 73.2%; at 2y=91.6%; at 3y=96.6% and over 99% of wounds healed from 4.9 years onwards.

***Bayesian meta-analysis***

For each included RCT in the MA, the most complete ulcer healing outcome data available were used: for one study time to ulcer healing and time to censoring were available as IPD (SWHSI-2); for the remaining RCTs, outcome data were reported as the number of healed ulcers by study group [aggregate data (AD)]. We maximally drew on all available data by statistically synthesising the AD with the IPD, extending the methodology described in Sutton et al 2010.^11^.

Data were synthesised by assuming that the time to healing underlying both AD and the IPD was described by the same parametric distribution. For the IPD, where time to healing (under censoring) was observed for each RCT participant – these data could directly inform the distribution of the time to healing (the likelihood). For the AD, by assuming a binomial likelihood, the number of participants healed was used to inform the probability of participants being healed. In turn, the probability of participants being healed was related (algebraically) to the common distribution of time to healing taking into account the duration of follow-up in each study. This approach allowed all ulcer healing data (proportion of ulcers healed and time to ulcer healing) to be defined as time to ulcer healing thus the measure of effectiveness used to report the MA findings was the HR.

Parametric survival modelling used: Log-normal (fitted to the IPD) with parameter sharing.

| **Bayesian MA synthesis modelling** | **FE MA ITT** | **RE MA ITT** | **FE MA, IPD covariate adjusted, ITT** | **RE MA, IPD covariate adjusted, ITT** |
| --- | --- | --- | --- | --- |
| **SWHSI-2 (IPD) and 6 AD** | | | | |
| **N** | 686 (SWHSI-2) + 784 (AD) | | 430 (SWHSI-2) + 784 (AD) | |
| **Pooled HR (median, 95%CrI)** | 1.02  (0.90 - 1.15) | 1.14  (0.85 - 1.83) | 1.04  (0.92 - 1.18) * | 1.07  (0.87 - 1.56) * |
| **DIC (measure goodness of fit)** | 2585.4 | 2586.3 | 1562.6 | 1563.4 |

MA, meta-analysis; FE, fixed-effects; RE, random-effects; AD, aggregate data; IPD, individual patient-level data; DIC, deviance information criteria; *area and treatment location are statistically significant.

Due to the small difference in the DIC statistic, the RE MA model covariate adjusted was chosen over the FE MA counterpart.

**Section 4 – Health-related Quality of Life in SWHSI-2 trial**

**Utility score by follow-up point and healing status**

| **EQ-5D utility score** | **N** | **Mean (SE)** | **95% CI** | |
| --- | --- | --- | --- | --- |
| **Unhealed utility: at month 0** | 656 | 0.607 (0.011) | 0.585 | 0.629 |
| **Unhealed utility: at month 3** | 339 | 0.624 (0.015) | 0.595 | 0.653 |
| **Unhealed utility: at month 6** | 193 | 0.607 (0.020) | 0.568 | 0.646 |
| **Unhealed utility: at month 12** | 96 | 0.591 (0.030) | 0.532 | 0.650 |
| **Healed utility: at month 0** | 0 | NA | NA | NA |
| **Healed utility: at month 3** | 66 | 0.695 (0.034) | 0.628 | 0.762 |
| **Healed utility: at month 6** | 189 | 0.709 (0.020) | 0.670 | 0.748 |
| **Healed utility: at month 12** | 253 | 0.658 (0.018) | 0.623 | 0.693 |

**Section 5 - Literature reviews for other economic model parameters**

***Search strategy***

Established cost-effectiveness, utilities, resource use and mortality search strategies,^12^ were used as a basis, with SWHSI and NPWT search terms from the Cochrane review also included to focus the population.^1^ The search for mortality was restricted to those studies assessing wound healing, and the resource use search was restricted to only UK settings. Initial searches were run in MEDLINE on 14^th^ June 2022 from date of database inception until the date of search. A further search was then conducted on 17^th^ October 2023, to identify any subsequent publications.

***Study Selection and Data Extraction***

Titles and abstracts retrieved from the searches were downloaded into Rayaan and de-duplicated.^13^ The remaining titles and abstracts were independently screened against pre-specified inclusion and exclusion criteria.

Studies were eligible for inclusion if they:

- Included patients with a SWHSI;
- Included NPWT as either the intervention or control treatment;
- Assessed cost-effectiveness, utilities, mortality, or resource use (as relevant to the search).

Studies were ineligible for inclusion if they:

- Were protocol, abstract or review publications. Where these were identified and potentially relevant, we sought to check that associated full report had not been omitted in the database search.

Full text copies were obtained for those articles deemed to be meeting inclusion criteria and these were again independently reviewed. Any disagreements or uncertainties were discussed and resolved between three reviewers. Using standardised data extraction forms for each search, data extraction was completed independently by two reviewers. Extraction by each reviewer was independently checked for consistency. No relevant studies were found that could appropriately inform the economic model.

**Section 6 - Unit costs**

**Table S2 - Unit costs of primary and secondary health care resources.**

| **Health resource** | **Unit cost** | **Description** | **Source** |
| --- | --- | --- | --- |
| GP surgery appointment | £41 | based on £41/9.22 minutes. 9.22-minute appointment | PSSRU 2023 [ref] |
| GP home appointment | £107.01 | based on £41/9.22 minutes. 11.4-minute home visit, 12 minutes of travel and 5 miles of travel assumed (£0.59 per mile) | PSSRU 2023 [ref] |
| Nurse surgery appointment | £13.43 | based on £52/h and 15.5 minutes of patient contact. Qualifications considered) | PSSRU 2023 [ref] |
| Nurse home appointment | £26.78 | based on £52/h and 15.5 minutes of patient contact, 12 minutes of travel and 5 miles of travel (£0.59/mile). Qualifications considered) | PSSRU 2023 [ref] |
| Hospital outpatient appointment (Diabetic Foot Clinic) | £183 | Service code: 307 (Diabetes Service) | NHS reference costs 2021/2022 |
| Hospital outpatient appointment (Podiatry) | £93 | Service code: 653 (Podiatry Service) | NHS reference costs 2021/2022 |
| Hospital outpatient appointment (Specialty Dressing Clinic) | £175 | Currency code: JB71A (Cleansing and Dressing of Burn, 16 years and over) | NHS reference costs 2021/2022 |
| Hospital outpatient appointment (Vascular, Colorectal or Plastics) | £176.33 | Average of service codes 104 (Colorectal Surgery Service), 107 (Vascular Surgery Service), 160 (Plastic Surgery Service) | NHS reference costs 2021/2022 |
| Hospital admission without overnight | £1,038.36 | Average cost of elective hospital stay | NHS reference costs 2021/2022 |
| Hospital inpatient nights (per night) | £492.19 | Elective inpatient excess bed-days (£431). Inflation adjusted according to World Bank GDP deflator for UK. | NHS reference costs 2017/2018 |
| Accident and emergency | £242 | Emergency care | NHS reference costs 2021/2022 |

**Table S3 - Unit costs of medication**

| **Medication** | **Cost / day** | **Description** | **Source** |
| --- | --- | --- | --- |
| Paracetamol | £0.19 | Paracetamol 500 mg caplets. 100 tablets: £2.34. Max use: 8 tablets per day | BNF (2023) [ref] |
| Co-codamol | £0.26 | Co-codamol 8mg/500mg, 100 tablets: £3.27. Max use: 8 tablets per day | BNF (2023) [ref] |
| Tramadol | £1.07 | Zydol 50mg soluble tablets, 100 tablets: £13.33 Max use: 8 tablets per day | BNF (2023) [ref] |
| Codeine Phosphate | £0.42 | Codeine 15mg tablets, 100 tablets: £2.64. Max use: 16 tablets per day | BNF (2023) [ref] |
| Short acting morphine | £0.51 | Morphine sulfate 20 mg tablets, 56 tablets: £9.50. Max use: Three 20 mg tablets per day | BNF (2023) [ref] |
| Long-acting morphine | £0.41 | Morphine sulfate 60 mg tablets, 60 tablets: £24.32. Max use: One 60mg tablet per day | BNF (2023) [ref] |
| Other morphine-based tablets | £0.45 | Kaolin and Morphine mixture; 200 ml: £2.27 Max use: 40 mL per day | BNF (2023) [ref] |
| Morphine oral suspension | £0.81 | Morphine sulfate 10mg/5ml oral solution.  500 ml: £6.72. Max use: 120mg per day, i.e. 60ml. | BNF (2023) [ref] |
| Amitriptyline | £0.07 | Amitriptyline 25mg tablets. 28 tablets: £0.68. Max use: 75 mg per day (neuropathic pain), i.e. 3 tablets per day. | BNF (2023) [ref] |
| Pregabalin | £0.34 | Pregabalin 75mg tablets. 56 tablets: £4.79. Initially 150 mg daily in 2–3 divided doses, then increased if necessary to 300 mg daily in 2–3 divided doses, dose to be increased after 3–7 days, then increased, if necessary, up to 600 mg daily in 2–3 divided doses, dose to be increased after 7 days. Assume 300mg per day, i.e. 4 tablets per day. | BNF (2023) [ref] |
| Gabapentin | £0.08 | Gabapentin 300mg capsules. 100 capsules: £2.55. Max use: 3 capsules (900mg) per day. | BNF (2023) [ref] |
| Diclofenac | £2.91 | Diclofenac .74 mg per 1 ml. 200mL: £12.95. Max use: 45 mL per day. | BNF (2023) [ref] |
| Aspirin | £1.63 | Aspirin 300mg tablets. 100 tablets: £12.56. Max use: 4g per day for pyrexia (by mouth), i.e. 13 tablets | BNF (2023) [ref] |
| Ibuprofen | £0.22 | Ibuprofen 200mg tablets. 250 tablets: £9.30. Max use: 1200mg per day, i.e. 6 tablets per day. | BNF (2023) [ref] |
| Antibiotics | £1.06 | Weighted average of:   - Flucloxacillin 500mg capsules. 28 capsules: £2.01. Max use: 2g/day, i.e. 4 tablets a day (£0.29) - CoAmoxiclav 500/125 mg capsules. 21 capsules. Max use: 500/125 mg every 8 hours for foot infection/leg ulcers, i.e. 3 tablets a day (£0.48) - Metronidazole 400mg tablets. Max use: 400 mg every 8 hours for leg ulcers, i.e. 3 tablets a day (£0.15) - Clindamycin 300 mg tablets. Max use: 300 mg every 6 hours for foot infection, i.e. 4 tablets a day (£4.60) - Ciprofloxacin. 500mg tablets. Max use: 500 mg twice daily, i.e. 2 tablets a day (£0.20) - Doxycycline. Max use: 100mg daily (200mg day 1 and then 100mg for another 6 days), i.e. 1 tablet a day on average (£0.61) | BNF (2023) [ref] |
| Beta Blockers | £0.03 | Amlodipine 5 mg 28 tablets. Max use: 5mg per day. | BNF (2023) [ref] |
| Anticoagulant | £0.18 | Apixaban 5mg 56 tablets. Max use: 10 mg twice daily, i.e. 2 tablets daily | BNF (2023) [ref] |
| Atorvastatin | £0.06 | Atorvastatin 20mg 28 tablets Max use: 20 mg once daily. | BNF (2023) [ref] |
| Clopidogrel | £0.04 | Clopidogrel 75mg 28 tablets. Max use: 75 mg once daily. | BNF (2023) [ref] |
| Analgesics (Oxycodone) | £8.94 | Oxycodone modified released medicine 40 mg 56 tablets-. Max use: 400mg per day, i.e. 10 tablets per day | BNF (2023) [ref] |
| Metformin | £0.02 | Metformin 28 tablets (500mg). Max use: 500mg per day. | BNF (2023) [ref] |
| Omeprazole | £0.21 | Omeprazole 28 tablets (20mg). Max use: 20mg per day. | BNF (2023) [ref] |
| Duloxetine | £0.31 | Duloxetine 28 tablets (60mg). Max dose: 60mg per day. | BNF (2023) [ref] |
| Isosorbide Mononitrate | £0.73 | Isosorbide Mononitrate 56 tablets (40mg). Max dose: 40mg twice per day. | BNF (2023) [ref] |
| Amlodipine | £0.03 | Amlodipine 28 tablets (10mg). Max dose: 10mg per day. | BNF (2023) [ref] |
| Perindopril | £0.04 | Perindopril erbumine 30 tablets (4mg). Max dose: 4mg per day. | BNF (2023) [ref] |

**Section 7 – Value of information analysis**

The maximum value of further research per patient treated, that is, the Expected Value of Perfect Information (EVPI), was derived from the difference between the Net Monetary Benefits (NMB) under perfect information (where the optimum treatment is known with certainty) and existing information (i.e. the expected net benefit from the treatment that is the most cost-effective under current state of knowledge). Intuitively, it is interpreted as the maximum amount that should be spent on research to resolve model uncertainty, given the potential impact, or benefit, of the research findings. The population-level EVPI represents the value of further research at population level and was estimated by multiplying the individual-level EVPI by the effective population size,^14^ as follows:

𝐸𝑓𝑓𝑒𝑐𝑡𝑖𝑣𝑒 𝑝𝑜𝑝𝑢𝑙𝑎𝑡𝑖𝑜𝑛 size = $\sum_{t=1}^{T} \frac{I_{t}}{{(1+d)}^{t}}$

Where It represents the incidence of surgical wounds in year t; T is the total number of years for which information from the research would be useful (usually representing the technology lifetime); and d is the discount rate, set at 3.5%.

In the VoI analysis, the yearly incidence of SWHSI in England and Wales was set to be approximately 410 per million.^15^ The relevant technology lifetime was assumed to be 5 years.

**Section 8 – Cost-effectiveness results**

**Figure S2 Cost-effectiveness acceptability curves – probability of treatments being cost-effective.**

**Figure S3 Population EVPI.**


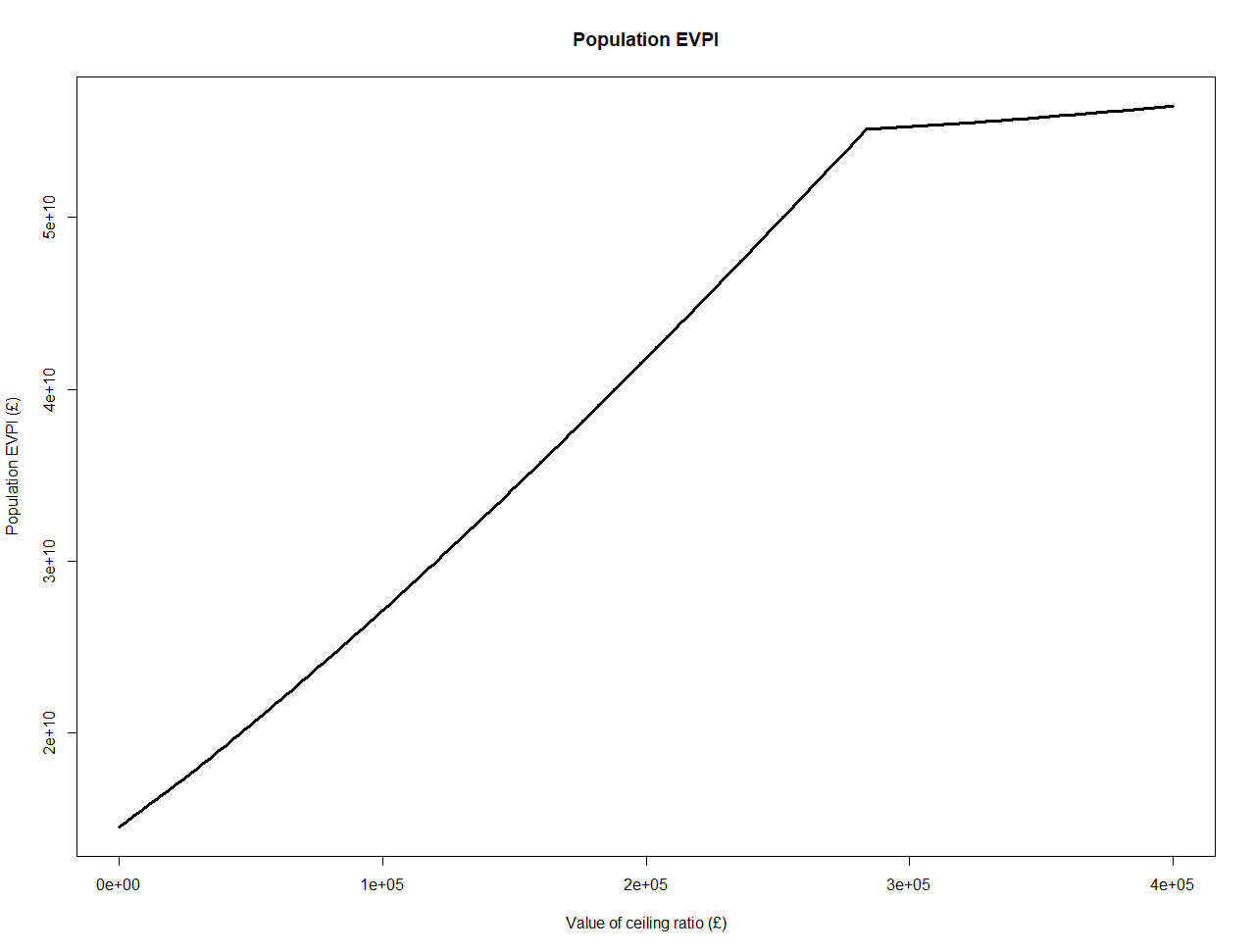


**Section 9 – Sensitivity/scenarios analysis over main cost-effectiveness results**

| **Scenario** | **Description** | **Characteristics** |
| --- | --- | --- |
| Scenario 1 | NPWT at any time vs non-NPWT (instead of ITT allocation) | NPWT =333 (192 (57.7%) healed over FU); NPWT median time to healing 203 days (95%CI, 174 - 238)  Usual care dressings =353 (206 (58.4%) healed over FU); Usual care dressings median time to healing 175 days (95%CI, 155 - 208) |
| Scenario 2a | Effectiveness from SWHSI-2 only ITT | HR: 1.00 (0.85 - 1.18) |
| Scenario 2b | Effectiveness from SWHSI-2 only NPWT at any time | HR: 1.07 (0.90 - 1.26) |
| Scenario 2c | Effectiveness from pooled HR with SWHSI-2 (only treatment adjusted) + 6 AD ITT | HR: 1.14 (0.84 - 1.82) |
| Scenario 2d | Effectiveness from pooled HR with SWHSI-2 (only treatment adjusted) + 6 AD NPWT at any time | HR: 1.13 (0.83 - 1.82) |
| Scenario 2e | Effectiveness from HR with SWHSI-2 only (only treatment adjusted) ITT | HR: 0.98 (0.84 - 1.15) |
| Scenario 2f | Effectiveness from HR with SWHSI-2 only (only treatment adjusted) NPWT at any time | HR: 1.06 (0.91 - 1.23) |
| Scenario 3 | Effectiveness as base case but different patient population (less severe population from SWHSI-1) | Descriptive statistics from SWHSI-1 (n=393), mean (SD):  Baseline age (years) = 54.1 (18.1)  Wound area (% >25cm2) = 23.4%  Treatment location (% inpatient (vs outpatient) = 57.5%  Tissue involvement (% skin and subcutaneous tissue loss (vs skin loss)) = 41.2%  SWHSI history (%) = 23.4% |
| Scenario 4a | Mortality adjustment from SWHSI-2 health state dependent | From healed to death, SMR=4.9  From unhealed to death, SMR=25.0 |
| Scenario 4b | Mortality adjustment from SWHSI-1 health state dependent | From healed to death, SMR=10.0  From unhealed to death, SMR=53.8 |
| Scenario 4c | Unadjusted mortality (i.e. mortality from the general population) | ONS 2022^16^ |
| Scenario 5 | Monthly utility decrement estimated from SWHSI-1 for patients in the unhealed wound health | Mean utility decrement: 0.018 (SE=0.007) |
| Scenario 6 | Health resource use from SWHSI-1 cohort | Average (SE) monthly health resources while unhealed (treatment dependent):  GP surgery appointment (NPWT): 1.20 (0.12)  GP surgery appointment (usual care): 0.70 (0.09)  GP home appointment (NPWT): 0.50 (0.11)  GP home appointment (usual care): 0.29 (0.03)  Nurse surgery appointment (NPWT): 4.26 (0.57)  Nurse surgery appointment (usual care): 2.52 (0.39)  Nurse home appointment (NPWT): 6.90 (0.55)  Nurse home appointment (usual care): 6.61 (0.70)  Average (SE) monthly health resources while unhealed (treatment independent):  Hospital outpatient appointment: 2.75 (0.37)  Hospital admission without overnight: 0.15 (0.06)  Hospital inpatient nights: 7.09 (0.90) |
| Scenario 7a | Time horizon of 1 year | Not applicable |
| Scenario 7b | Time horizon of 5 years | Not applicable |

**Effectiveness - Scenario 1 – Treatment allocation as NPWT at any time of the follow-up *versus* non-NPWT**

| **Scenario 1 - Mean (95% CI)** | **NPWT (at any time)** | | **Usual Care (non-NPWT)** | |
| --- | --- | --- | --- | --- |
| Total QALYs | 3.77 | (3.63 – 3.86) | 3.78 | (3.65 – 3.87) |
| Total costs (£) | 69,084 | (52,696 – 87,020) | 68,052 | (54,183 – 84,544) |
| Health care costs | 51,048 | (36,711 – 67,516) | 54,532 | (41,020 – 70,329) |
| Intervention costs | 18,036 | (14,350 – 21,685) | 13,521 | (12,006 – 15,035) |
| Incremental costs (vs UC) (£) | 1,031 (-10,079 – 10,231) | | | |
| Incremental QALYs (vs UC) | 0.02 (-0.02 – 0.06) | | | |
| k=£20,000/QALY gained (£) |  | | | |
| NMB | 6,521 (-12,052 – 23,390) | | 7,249 (-8,367 – 21,668) | |
| INMB | -728 (-10,419 – 10,860) | | | |
| Probability NPWT cost-effective | 40.8% | | | |
| k=£30,000/QALY gained (£) |  | | | |
| NMB | 44,323 (25,228 – 61,440) | | 44,899 (28,840 – 59,701) | |
| INMB | -576 (-10,588 – 11,276) | | | |
| Probability NPWT cost-effective | 41.5% | | | |

**Effectiveness - Scenario 2a – Effectiveness from SWHSI-2 only ITT, HR: 1.00 (0.85 to 1.18)**

| **Mean (95% CI)** | **NPWT** | | **Usual Care** | |
| --- | --- | --- | --- | --- |
| Total QALYs | 3.84 | (3.78 – 3.88) | 3.83 | (3.78 – 3.88) |
| Total costs (£) | 47,188 | (37,566 – 58,621) | 41,427 | (33,914 – 50,278) |
| Health care costs | 33,516 | (25,263 – 41,882) | 33,220 | (25,860 – 41,882) |
| Intervention costs | 13,672 | (10,627 – 16,763) | 8,207 | (7,760 – 8,661) |
| Incremental costs (vs UC) (£) | 5,761 (-563 – 12,259) | | | |
| Incremental QALYs (vs UC) | -0.0003 (-0.01 – 0.01) | | | |
| k=£20,000/QALY gained (£) |  | | | |
| NMB | 29,592 (18,200 – 39,473) | | 35,359 (26,658 – 42,813) | |
| INMB | -5,766 (-12,365 – 723) | | | |
| Probability NPWT cost-effective | 4.7% | | | |
| k=£30,000/QALY gained (£) |  | | | |
| NMB | 67,983 (56,342 – 77,954) | | 73,752 (64,816 – 81,289) | |
| INMB | -5,769 (-12,412 – 808) | | | |
| Probability NPWT cost-effective | 5.1% | | | |

**Effectiveness - Scenario 2b – Effectiveness from SWHSI-2 only NPWT ever, HR: 1.07 (0.90 to 1.26)**

| **Mean (95% CI)** | **NPWT** | | **Usual Care** | |
| --- | --- | --- | --- | --- |
| Total QALYs | 3.84 | (3.77 – 3.88) | 3.84 | (3.78 – 3.88) |
| Total costs (£) | 49,191 | (39,166 – 61,243) | 41,220 | (33,753 – 50,036) |
| Health care costs | 35,140 | (26,492 – 45,962) | 33,054 | (25,725 – 41,685) |
| Intervention costs | 14,051 | (10,964 – 17,181) | 8,166 | (7,724 – 8,620) |
| Incremental costs (vs UC) (£) | 7,971 (1,468 – 14,720) | | | |
| Incremental QALYs (vs UC) | -0.004 (-0.02 – 0.01) | | | |
| k=£20,000/QALY gained (£) |  | | | |
| NMB | 27,523 (15,635 – 37,764) | | 35,573 (26,932 – 43,005) | |
| INMB | -8,050 (-14,927 – -1,371) | | | |
| Probability NPWT cost-effective | 1.1% | | | |
| k=£30,000/QALY gained (£) |  | | | |
| NMB | 65,879 (53,740 – 76,218) | | 73,970 (65,086 – 81,466) | |
| INMB | -8,090 (-15,039 – -1,320) | | | |
| Probability NPWT cost-effective | 1.1% | | | |

**Effectiveness Scenario 2c – Effectiveness from pooled HR with SWHSI-2 (only treatment adjusted) + 6 AD ITT, HR: 1.14 [0.84 to 1.82]**

| **Mean (95% CI)** | **NPWT** | | **Usual Care** | |
| --- | --- | --- | --- | --- |
| Total QALYs | 3.80 | (3.69 – 3.87) | 3.80 | (3.69 – 3.87) |
| Total costs (£) | 62,415 | (48,569 – 77,789) | 59,002 | (47,356 – 72,455) |
| Health care costs | 45,743 | (33,720 – 60,194) | 47,282 | (35,937 – 60,686) |
| Intervention costs | 16,672 | (13,462 – 19,705) | 11,721 | (10,805 – 12,617) |
| Incremental costs (vs UC) (£) | 3,413 (-4,594 – 10,572) | | | |
| Incremental QALYs (vs UC) | 0.006 (-0.02 – 0.03) | | | |
| k=£20,000/QALY gained (£) |  | | | |
| NMB | 13,640 (-1,527 – 28,028) | | 16,929 (3,194 – 29,146) | |
| INMB | -3,286 (-10,818 – 5,059) | | | |
| Probability NPWT cost-effective | 22.3% | | | |
| k=£30,000/QALY gained (£) |  | | | |
| NMB | 51,668 (36,215 – 66,187) | | 54,895 (40,776 – 67,280) | |
| INMB | -3,224 (-10,961 – 5,335) | | | |
| Probability NPWT cost-effective | 22.3% | | | |

**Effectiveness Scenario 2d – Effectiveness from pooled HR with SWHSI-2 (only treatment adjusted) + 6 AD NPWT ever, HR: 1.13 [0.83 to 1.82]**

| **Mean (95% CI)** | **NPWT** | | **Usual Care** | |
| --- | --- | --- | --- | --- |
| Total QALYs | 3.81 | (3.72 – 3.87) | 3.80 | (3.70 – 3.87) |
| Total costs (£) | 59,872 | (46,864 – 77,144) | 58,284 | (46,066 – 71,343) |
| Health care costs | 43,717 | (31,759 – 59,097) | 46,704 | (35,037 – 59,379) |
| Intervention costs | 16,154 | (13,000 – 19,594) | 11,580 | (10,621 – 12,547) |
| Incremental costs (vs UC) (£) | 1,587 (-6,538 – 11,815) | | | |
| Incremental QALYs (vs UC) | 0.01 (-0.02 – 0.04) | | | |
| k=£20,000/QALY gained (£) |  | | | |
| NMB | 16,329 (-1,003 – 29,676) | | 17,676 (4,315 – 30,029) | |
| INMB | -1,319 (-12,253 – 7,040) | | | |
| Probability NPWT cost-effective | 42.0% | | | |
| k=£30,000/QALY gained (£) |  | | | |
| NMB | 54,429 (36,814 – 67,876) | | 55,656 (41,983 – 68,070) | |
| INMB | -1,199 (-12,424 – 7,306) | | | |
| Probability NPWT cost-effective | 43.1% | | | |

**Effectiveness Scenario 2e – Effectiveness from HR with SWHSI-2 only (only treatment adjusted) ITT, HR: 0.98 [0.84 to 1.15]**

| **Mean (95% CI)** | **NPWT** | | **Usual Care** | |
| --- | --- | --- | --- | --- |
| Total QALYs | 3.83 | (3.76 – 3.88) | 3.83 | (3.77 – 3.88) |
| Total costs (£) | 49,522 | (39,554 – 60,344) | 44,570 | (35,927 – 54,094) |
| Health care costs | 35,396 | (26,349 – 46,305) | 35,747 | (27,065 – 45,218) |
| Intervention costs | 14,127 | (11,045 – 16,906) | 8,823 | (8,427 – 9,229) |
| Incremental costs (vs UC) (£) | 4,952 (-700 – 10,743) | | | |
| Incremental QALYs (vs UC) | 0.001 (-0.01 – 0.01) | | | |
| k=£20,000/QALY gained (£) |  | | | |
| NMB | 27,153 (16,024 – 37,430) | | 32,087 (22,441 – 41,186) | |
| INMB | -4,934 (-10,847 – 888) | | | |
| Probability NPWT cost-effective | 4.6% | | | |
| k=£30,000/QALY gained (£) |  | | | |
| NMB | 65,490 (54,173 – 75,814) | | 70,415 (60,758 – 79,634) | |
| INMB | -4,925 (-10,899 – 1,018) | | | |
| Probability NPWT cost-effective | 5.3% | | | |

**Effectiveness Scenario 2f – Effectiveness from HR with SWHSI-2 only (only treatment adjusted) NPWT ever, HR: 1.06 [0.91 to 1.23]**

| **Mean (95% CI)** | **NPWT** | | **Usual Care** | |
| --- | --- | --- | --- | --- |
| Total QALYs | 3.83 | (3.76 – 3.88) | 3.83 | (3.77 – 3.88) |
| Total costs (£) | 51,685 | (41,305 – 63,113) | 44,339 | (35,737 – 53,838) |
| Health care costs | 37,149 | (27,658 – 48,581) | 35,561 | (26,919 – 44,990) |
| Intervention costs | 14,535 | (11,464 – 17,329) | 8,778 | (8,382 – 9,184) |
| Incremental costs (vs UC) (£) | 7,346 (1,562 – 13,510) | | | |
| Incremental QALYs (vs UC) | -0.003 (-0.02 – 0.01) | | | |
| k=£20,000/QALY gained (£) |  | | | |
| NMB | 24,914 (13,269 – 35,590) | | 32,326 (22,750 – 41,378) | |
| INMB | -7,412 (-13,715 – 1,407) | | | |
| Probability NPWT cost-effective | 0.8% | | | |
| k=£30,000/QALY gained (£) |  | | | |
| NMB | 63,213 (51,362 – 73,930) | | 70,659 (61,057 – 79,805) | |
| INMB | -7,446 (-13,817 – 1,338) | | | |
| Probability NPWT cost-effective | 0.8% | | | |

**Scenario 3 - Effectiveness as base case but different patient population (less severe population from SWHSI-1)**

| **Mean (95% CI)** | **NPWT** | | **Usual Care** | |
| --- | --- | --- | --- | --- |
| Total QALYs | 6.14 | (5.97 – 6.24) | 6.12 | (5.96 – 6.23) |
| Total costs (£) | 70,993 | (51,473 – 90,808) | 69,015 | (53,918 – 85,241) |
| Health care costs | 52,548 | (36,564 – 70,408) | 55,299 | (41,102 – 70,491) |
| Intervention costs | 18,445 | (14,026 – 22,285) | 13,715 | (11,909 – 15,571) |
| Incremental costs (vs UC) (£) | 1,979 (-12,236 – 11,997) | | | |
| Incremental QALYs (vs UC) | 0.01 (-0.03 – 0.07) | | | |
| k=£20,000/QALY gained (£) |  | | | |
| NMB | 51,748 (31,014 – 72,080) | | 53,468 (36,788 – 69,320) | |
| INMB | -1,720 (-12,987 – 13,079) | | | |
| Probability NPWT cost-effective | 32.5% | | | |
| k=£30,000/QALY gained (£) |  | | | |
| NMB | 113,118 (92,199 – 133,709) | | 114,709 (97,958 – 131,447) | |
| INMB | -1,590 (-13,116 – 13,630) | | | |
| Probability NPWT cost-effective | 33.1% | | | |

**Scenario 4a - Mortality adjustment from SWHSI-2 health state dependent: Healed SMR=4.9; Unhealed SMR=25.0**

| **Mean (95% CI)** | **NPWT** | | **Usual Care** | |
| --- | --- | --- | --- | --- |
| Total QALYs | 5.36 | (5.04 – 5.70) | 5.28 | (5.04 – 5.48) |
| Total costs (£) | 65,875 | (51,344 – 80,893) | 62,572 | (49,940 – 75,729) |
| Health care costs | 48,455 | (35,550 – 62,672) | 50,138 | (37,603 – 63,393) |
| Intervention costs | 17,419 | (13,866 – 20,591) | 12,434 | (11,352 – 13,542) |
| Incremental costs (vs UC) (£) | 3,302 (-5,621 – 9,926) | | | |
| Incremental QALYs (vs UC) | 0.08 (-0.14 – 0.37) | | | |
| k=£20,000/QALY gained (£) |  | | | |
| NMB | 41,336 (22,576 – 61,030) | | 43,108 (27,931 – 57,878) | |
| INMB | -1,772 (-12,670 – 13,256) | | | |
| Probability NPWT cost-effective | 32.0% | | | |
| k=£30,000/QALY gained (£) |  | | | |
| NMB | 94,941 (74,283 – 117,766) | | 95,948 (79,700 – 111,914) | |
| INMB | -1,007 (-14,032 – 17,258) | | | |
| Probability NPWT cost-effective | 37.3% | | | |

**Scenario 4b - Mortality adjustment from SWHSI-1 health state dependent: Healed SMR=10.0; Unhealed SMR=53.8**

| **Mean (95% CI)** | **NPWT** | | **Usual Care** | |
| --- | --- | --- | --- | --- |
| Total QALYs | 3.31 | (3.08 – 3.56) | 3.25 | (3.08 – 3.40) |
| Total costs (£) | 55,860 | (44,436 – 67,930) | 51,666 | (41,286 – 62,501) |
| Health care costs | 40,399 | (30,256 – 51,925) | 41,400 | (31,215 – 51,967) |
| Intervention costs | 15,461 | (12,297 – 18,429) | 10,267 | (9,579 – 10,979) |
| Incremental costs (vs UC) (£) | 4,193 (-1,768 – 8,785) | | | |
| Incremental QALYs (vs UC) | 0.06 (-0.10 – 0.27) | | | |
| k=£20,000/QALY gained (£) |  | | | |
| NMB | 10,332 (-3,429 – 24,677) | | 13,424 (1,672 – 25,041) | |
| INMB | -3,092 (-10,347 – 6,916) | | | |
| Probability NPWT cost-effective | 19.6% | | | |
| k=£30,000/QALY gained (£) |  | | | |
| NMB | 43,429 (28,167 – 59,927) | | 45,969 (33,599 – 58,026) | |
| INMB | -2,541 (-11,565 – 9,681) | | | |
| Probability NPWT cost-effective | 25.4% | | | |

**Scenario 4c - Unadjusted mortality (i.e. from the general population)**

| **Mean (95% CI)** | **NPWT** | | **Usual Care** | |
| --- | --- | --- | --- | --- |
| Total QALYs | 10.73 | (10.51 – 10.86) | 10.71 | (10.49 – 10.85) |
| Total costs (£) | 79,822 | (59,191 – 100,991) | 78,107 | (61,019 – 96,529) |
| Health care costs | 59,656 | (42,127 – 79,262) | 62,585 | (46,833 – 80,063) |
| Intervention costs | 20,166 | (15,784 – 23,962) | 15,522 | (13,700 – 17,429) |
| Incremental costs (vs UC) (£) | 1,715 (-13,199 – 12,418) | | | |
| Incremental QALYs (vs UC) | 0.02 (-0.05 – 0.11) | | | |
| k=£20,000/QALY gained (£) |  | | | |
| NMB | 134,755 (112,346 – 157,159) | | 136,085 (117,511 – 154,006) | |
| INMB | -1,330 (-13,555 – 14,738) | | | |
| Probability NPWT cost-effective | 35.2% | | | |
| k=£30,000/QALY gained (£) |  | | | |
| NMB | 242,043 (218,880 – 265,135) | | 243,180 (223,719 – 261,963) | |
| INMB | -1,137 (-13,998 – 15,528) | | | |
| Probability NPWT cost-effective | 36.7% | | | |

**Scenario 5 - Monthly utility decrement estimated from SWHSI-1 for patients in the unhealed wound health state of 0.018 (0.007)**

| **Mean (95% CI)** | **NPWT** | | **Usual Care** | |
| --- | --- | --- | --- | --- |
| Total QALYs | 3.66 | (3.43 – 3.81) | 3.64 | (3.41 – 3.80) |
| Total costs (£) | 71,650 | (55,047 – 88,756) | 69,925 | (54,874 – 84,589) |
| Health care costs | 53,077 | (38,546 – 68,707) | 55,223 | (41,685 – 69,930) |
| Intervention costs | 18,573 | (15,170 – 21,939) | 13,702 | (12,349 – 15,101) |
| Incremental costs (vs UC) (£) | 2,725 (-8,391 – 10,914) | | | |
| Incremental QALYs (vs UC) | 0.02 (-0.04 – 0.10) | | | |
| k=£20,000/QALY gained (£) |  | | | |
| NMB | 1,451 (-17,417 – 19,282) | | 3,811 (-13,137 – 18,475) | |
| INMB | -2,361 (-11,335 – 10,171) | | | |
| Probability NPWT cost-effective | 27.0% | | | |
| k=£30,000/QALY gained (£) |  | | | |
| NMB | 38,001 (18,198 – 56,211) | | 40,179 (22,898 – 55,311) | |
| INMB | -2,178 (-11,933 – 11,323) | | | |
| Probability NPWT cost-effective | 28.7% | | | |

**Scenario 6 - Health resource use from SWHSI-1 cohort**

| **Mean (95% CI)** | **NPWT** | | **Usual Care** | |
| --- | --- | --- | --- | --- |
| Total QALYs | 3.77 | (3.63 – 3.86) | 3.76 | (3.62 – 3.86) |
| Total costs (£) | 488,600 | (364,448 – 622,633) | 503,079 | (402,289 – 617,077) |
| Incremental costs (vs UC) (£) | -14,480 (-95,176 – 43,555) | | | |
| Incremental QALYs (vs UC) | 0.01 (-0.02 – 0.05) | | | |
| k=£20,000/QALY gained (£) |  | | | |
| NMB | -413,176 (-547,267 – -291,137) | | -427,846 (-541,570 – -326,925) | |
| INMB | 14,670 (-44,008 – 95,610) | | | |
| Probability NPWT cost-effective | 64.5% | | | |
| k=£30,000/QALY gained (£) |  | | | |
| NMB | -375,465 (-509,569 – -254,483) | | -390,229 (-503,699 – -289,260) | |
| INMB | 14,765 (-44,216 – 95,827) | | | |
| Probability NPWT cost-effective | 64.5% | | | |

**Scenario 7a - Time Horizon = 1 year**

| **Mean (95% CI)** | **NPWT** | | **Usual Care** | |
| --- | --- | --- | --- | --- |
| Total QALYs | 0.69 | (0.62 – 0.74) | 0.69 | (0.62 – 0.74) |
| Total costs (£) | 52,273 | (42,731 – 62,415) | 47,312 | (38,363 – 57,049) |
| Health care costs | 37,571 | (28,728 – 47,800) | 37,911 | (29,090 – 47,800) |
| Intervention costs | 14,702 | (11,852 – 17,267) | 9,401 | (8,997 – 9,756) |
| Incremental costs (vs UC) (£) | 4,960 (1,452 – 8,195) | | | |
| Incremental QALYs (vs UC) | 0.001 (-0.002 – 0.01) | | | |
| k=£20,000/QALY gained (£) |  | | | |
| NMB | -38,421 (-48,387 – -28,639) | | -33,479 (-43,015 – -24,634) | |
| INMB | -4,942 (-8,190 – -1,415) | | | |
| Probability NPWT cost-effective | 0.3% | | | |
| k=£30,000/QALY gained (£) |  | | | |
| NMB | -31,495 (-41,438 – -21,616) | | -26,563 (-36,145 – -17,733) | |
| INMB | -4,933 (-8,183 – -1,383) | | | |
| Probability NPWT cost-effective | 0.3% | | | |

**Scenario 7b - Time Horizon = 5 year**

| **Mean (95% CI)** | **NPWT** | | **Usual Care** | |
| --- | --- | --- | --- | --- |
| Total QALYs | 2.63 | (2.50 – 2.71) | 2.62 | (2.49 – 2.71) |
| Total costs (£) | 71,092 | (54,715 – 87,709) | 68,261 | (54,268 – 83,180) |
| Health care costs | 52,648 | (38,317 – 68,581) | 54,696 | (40,943 – 68,973) |
| Intervention costs | 18,443 | (14,725 – 21,787) | 13,565 | (12,276 – 14,879) |
| Incremental costs (vs UC) (£) | 2,831 (-7,963 – 10,453) | | | |
| Incremental QALYs (vs UC) | 0.01 (-0.02 – 0.04) | | | |
| k=£20,000/QALY gained (£) |  | | | |
| NMB | -18,539 (-35,496 – -1,335) | | -15,858 (-30,914 – -1,321) | |
| INMB | -2,682 (-10,779 – 8,743) | | | |
| Probability NPWT cost-effective | 23.5% | | | |
| k=£30,000/QALY gained (£) |  | | | |
| NMB | 7,737 (-9,692 – 25,245) | | 10,344 (-4,584 – 25,136) | |
| INMB | -2,607 (-11,040 – 9,058) | | | |
| Probability NPWT cost-effective | 24.0% | | | |

**References**

1. Dumville JC., Owens GL., Crosbie EJ., et al. Negative pressure wound therapy for treating surgical wounds healing by secondary intention. *Cochrane Database Syst Rev* 2015; 4. DOI: 10.1002/14651858.CD011278.pub2.

2. Saramago P, Chuang L and Soares MO. Network meta-analysis of (individual patient) time to event data alongside (aggregate) count data. *BMC Med Res Methodol* 2014; 14: 105. DOI: <https://doi.org/10.1186/1471-2288-14-105>

3. Speigelhalter DJ., Best NG., Carlin BP. and Van der Linde A. Bayesian Measures of Model Complexity and Fit. *Journal of the Royal Statistical Society Series B: Statistical Methodology* 2002; 64: 583-639. DOI: <https://doi.org/10.1111/1467-9868.00353>

4. Acosta S., Monsen C. and Dencker M. Clinical outcome and microvascular blood flow in VAC - and Sorbalgon - treated peri-vascular infected wounds in the groin after vascular surgery – an early interim analysis. *Int Wound J* 2012; 10: 377-382. DOI: <https://doi.org/10.1111/j.1742-481X.2012.00993.x>.

5. Armstrong DG. and Lavery LA. Negative pressure wound therapy after partial diabetic foot amputation: a multicentre, randomised controlled trial. *Lancet* 2005; 366: 1704-1710.

6. Ulas-Biter L., Beck GMN., Mannaerts GHH., et al. The use of negative-pressure wound therapy in pilonidal sinus disease: a randomized controlled trial comparing negative-pressure wound therapy versus standard open wound care after surgical excision. *Dis Colon Rectum* 2014; 57.

7. Chiang N., Rodda OA., Sleigh J. and Vasudevan T. Effects of topical negative pressure therapy on tissue oxygenation and wound healing in vascular foot wounds. *J Vasc Surg* 2017; 66: 564-571. DOI: <https://doi.org/10.1016/j.jvs.2017.02.050>.

8. Monsen C., Wann-Hansson C., Wictorsson C. and Acosta S. Vacuum-assisted wound closure versus alginate for the treatment of deep perivascular wound infections in the groin after vascular surgery. *J Vasc Surg* 2014; 59: 145-151.

9. Seidel D., Diedrich S., Herrle F., et al. Negative Pressure Wound Therapy vs Conventional Wound Treatment in Subcutaneous Abdominal Wound Healing Impairment: The SAWHI Randomized Clinical Trial. *JAMA Surgery* 2020; 155: 469-478. DOI: <https://doi.org/10.1001/jamasurg.2020.0414>.

10. Sterne J, Savovic J, Page M, et al. RoB 2: a revised tool for assessing risk of bias in randomised trials. . *British Medical Journal* 2019; 366: I4898. DOI: <https://doi.org/10.1136/bmj.l4898>.

11. Sutton A., Kendrick D. and Coupland C. Meta-analysis of individual- and aggregate-level data. *Stat Med* 2008; 27: 651-669. DOI: <https://doi.org/10.1002/sim.2916>.

12. Ashby RL. GR, Ali S, Saramago P, Chuang LH, Adderley U et al. VenUS IV (Venous leg Ulcer Study IV) - compression hosiery compared with compression bandaging in the treatment of venous leg ulcers: a randomised controlled trial, mixed-treatment comparison and decision-analytic model. *Health Technol Assess* 2014; 18: 1-293.

13. Ouzzani M., Hammady H., Fedorowicz Z. and Elmagarmid A. Rayyan — a web and mobile app for systematic reviews. *Systematic Reviews* 2016; 5. DOI: <https://doi.org/10.1186/s13643-016-0384-4>.

14. Claxton KP. and Sculpher MJ. Using value of information analysis to prioritise health research: Some lessons from recent UK experience. . *PharmacoEconomics* 2006; 24: 1055-1068. DOI: <https://doi.org/10.2165/00019053-200624110-00003>.

15. Chetter IC., Oswald AV., McGinnis E., et al. Patients with surgical wounds healing by secondary intention: A prospective, cohort study. *Int J Nurs Stud* 2019; 89: 62-71. DOI: <https://doi.org/10.1016/j.ijnurstu.2018.09.011>.

16. Office for National Statistics. 21st Century Mortality dataset, England and Wales, 2000 to 2022. In: Office for National Statistics., (ed.). United Kingdom2023.
